# Application of a system dynamics model in forecasting the supply and age distribution of physicians

**DOI:** 10.3325/cmj.2020.61.100

**Published:** 2020-04

**Authors:** Danko Relić, Jadranka Božikov

**Affiliations:** 1 Department of Medical Statistics, Epidemiology and Medical Informatics, Andrija Štampar School of Public Health, University of Zagreb School of Medicine, Zagreb, Croatia; 2 Center for Career Planning in Biomedicine and Health, University of Zagreb School of Medicine, Zagreb, Croatia

## Abstract

**Aim:**

To predict the future supply and age distribution of physicians with a simulation model, which can be used as an advising tool for policymakers who decide on enrollment and specialization training (ST) quotas at the national level.

**Methods:**

A simulation model was created using the system dynamics (SD) method. Changes in the number of physicians and their age distribution were projected in the context of the expected future changes of the Croatian population under different scenarios covering the period from 2017 to 2041.

**Results:**

The two scenarios showed that Croatia would not face physician shortage in the future. The scenario 1 projected that Croatia would certainly reach the current European Union (EU) average of 360 physicians per 100 000 inhabitants by 2021, and that this figure would increase to 430 per 100 000 inhabitants by 2041. The scenario 2 suggested a similar trend, with Croatia reaching the current EU average by 2021 and the number of physicians increasing to 451 per 100 000 inhabitants by 2041. Both scenarios indicated that the Croatian physicians’ age distribution will recover in favor of younger age groups of specialists.

**Conclusion:**

There is no need to increase the medical school enrollment to ensure sufficient number of physicians per capita in Croatia, but it is necessary to keep the recently reached level of 550 licenses for ST per year. The developed dynamic model is available online and can be adapted to the analysis of different scenarios in different health care systems.

The human population is aging: the number of older people is increasing in almost all countries. This affects all segments of society, including labor and financial markets (1). Population aging has a particular impact on health care, since it affects not only those who we care for but also those who care for others – health care workforce.

According to the United Nations reports, the percentage of people older than 60 in Croatia is 26.8, which makes it the seventh country in the world in terms of population age. At the top of the list is Japan with 33.5% and Italy with 29.4% (1).

According to the mid-2018 estimate, Croatia had 4 087 843 inhabitants. As compared with the previous year, the population decreased by 36 688 persons or 0.9%, largely due to emigration (2). Moreover, from 2007 to 2016, the population decreased by 4% or almost 160 000 people. This decrease, however, was not uniform in all age groups. The only demographic increase was recorded in the older age groups, which are the largest beneficiary of medical services and health care.

Following Croatia’s accession to the European Union (EU) in 2013, the emigration of health workers to other Member States has increased. The majority of physicians are concentrated in major cities, and their density varies among the country's 21 counties. In some counties (mostly the less developed ones) over one-third of physicians are aged 55 and over. This raises concerns about the number of physicians in the near future. The average age of practicing physicians employed in the health sector in Croatia is 49.7 years, with most of them being 55 to 59 years old, followed by those who are 50 to 54 years old (3). This is in accordance with trends in the EU, where the share of physicians aged 55 or over rose from 27% to 38% between 2005 and 2016 (4).

During the last decade (2007-2016), the supply of physicians has grown faster than the population (3). The need for physicians is expected to increase as the share of the elderly population and the incidence of chronic diseases requiring intensified medical care increase. At the same time, with a large number of physicians approaching the retirement age, it will be necessary to carefully plan human resources for health, with particular emphasis on deficit health care activities and deficit regions of Croatia.

Simulation models have been successfully used in research, instruction, and training, as well as to aid the decision-making processes. This is why they have been also known as “policy simulations.” They allow us to emulate complex systems behavior in situations when experiments cannot be performed as well as to predict future events under simulated circumstances. The main advantage of policy simulations is their ability to answer the “What if …?” type of questions (5).

The prediction of future events based on present data are the basis of all business planning and forecasting, including the planning of human resources for health needs and supply. Different forecasting techniques are in use, from those based on statistical methods to simulation models. Some of them have also been applied in Croatia (6). There are several methods for planning and projecting human resources for health (7), including regression-based models, simulation models (8-11), and Markov chains (12). The principles of system dynamics (SD) originated 60 years ago followed by the development of the software used for simulation modeling of all aspects of industrial systems and urban growth. There is a large body of literature describing both methodological fundamentals and applications of these models (13). An SD model of a future number of physicians was designed and implemented in Croatia almost three decades ago, when the country was facing a surplus of physicians (14,15). The results of this simulation were used as an input in the process of reducing medical school enrollment from 600-700 to 450-500. This was finally implemented from 1993 onwards.

The aim of the current study is to use a simulation model to predict the future supply and age distribution of physicians. The model is designed as an advising tool for policymakers who decide on enrollment and specialization training (ST) quotas at the national level.

## Methods

System dynamics (SD) method represents the real system in the form of causal loop diagrams and flowcharts by means of Stella Architect ver. 1.9.2 (isee systems, Lebanon, NH, USA), a specialized modeling software package (16).

**Figure 1:**
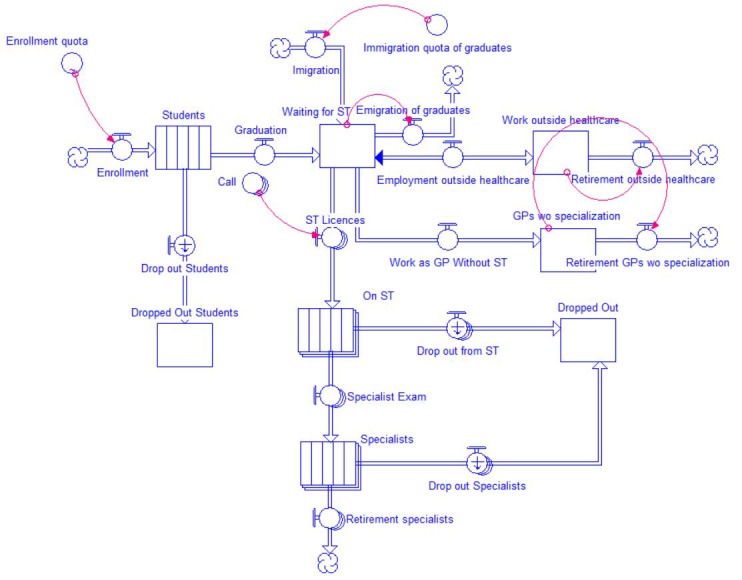
Diagram of the system dynamics model for forecasting the number of physicians with age distribution.

Two different scenarios were simulated. The prediction was generated through the model shown in Figure 1, with parameters and initial values based on data from public institutions in Croatia shown in Table 1. The baseline year was 2017, and projections were made for the future until 2041. The model is available at *https://exchange.iseesystems.com/models/player/jadrankabozikov/prototip-ageing-5120*(It can be run either online or after download. Readers' feedback is highly appreciated).

**Table 1:** The main parameters of the model

Inflow (persons/year)	Professional stage – initial values in 2017 (Current number of persons)	Outflow
Enrollment* Scenario 1: 590 → Scenario 2: 590 →	Medical schools (3761 students)* **Duration: 6 y**	→ 10% of generation^†‡^
Approximately 530 from medical schools^‡^ → 20 immigrants^§^ →	**Waiting for ST (1246 MDs)^‖^**	→ Emigration 5% per year^‡^ → 20 persons/y^¶‡^
**Licenses for ST** Scenario 1: 450 → Scenario 2: 550 from 2020 →	**On ST (2268 MDs)** **Duration: 5 y^‖^**	→ 0.2% per year**†^‡^
**All specialists**	**Specialists (9648)^‖^**	→ 1% per year**^‡^
	**Working as GPs without specialization (1232 MDs)^‖^**	→ Retirement in next 20 y^††^
	**MDs working outside health care (1200 MDs)^‡^**	→ Retirement

ST – specialization training, MD – medical doctor, GP – general practitioner.

*Croatian Medical Schools.

†Drop-out from medical schools.

‡Estimation.

§Government decision valid for 2017.

**‖**Croatian Medical Chamber (3).

¶Employment outside the health care system.

**drop-out due to emigration, disability, and mortality;

††Legal requirements on retirement age.

Following the international recommendations and practices of most developed countries, the term physician refers only to medical doctors directly providing health care services to patients.

Medical schools in collaboration with the Ministry of Science and Education determine the number of enrolled students. Drop out of students from medical schools is approximately 10%. The Ministry of Health announces calls for ST based on the expressed needs of health care institutions. The duration of ST is defined by the Act on Specialization Training of Physicians in Croatia issued by the Ministry of Health – most of them last 5 years.

Population predictions for 2021, 2031, and 2041 were obtained from studies and projections of prominent Croatian demographic experts (17). These projections were shown to be much more accurate than the projections of the Croatian Central Bureau of Statistics.

Although residency is mandatory today, this was not the case in the past. As a consequence, a certain number of physicians permanently work as general practitioners (GPs) without residency and will retire as such. Most physicians become specialists at the around 30 years, meaning that their remaining working life is 35 years, although in 2038 the retirement age will be extended to 67 years.

**Figure 2:**
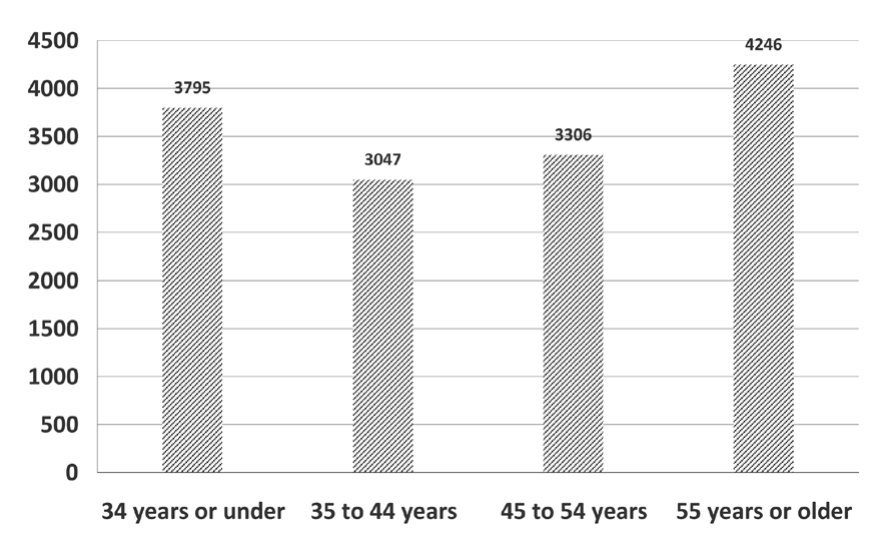
Age distribution of Croatian physicians (2016).

A total of 29.5% of physicians are in the age group 55 years and older (Figure 2). Having in mind that according to present regulations the retirement age is 65 years, almost 30% of physicians will retire from the workforce in the next 10 years (18).

### SD model validation

It is important to check the validity of the simulation model. Available historical data were used for model validation and compared with simulation results. The model is considered reliable if the relative error is less than 0.1 (9). The analysis of sensitivity to main input parameters showed that every change in one of input parameters yielded the same change in output variables (ie, if the number of realized licenses for ST increases/decreases for 5%, the output variable in the long run will show an equal increase/decrease).

## Results

The simulated data were compared with historical data, and the relative errors were less than 0.1 (Table 2). Several scenarios were simulated with different model parameters and input values. Two most realistic and desirable scenarios are presented. The major elements in the model are defined by the professional stage (study of medicine, time of waiting for ST, time on ST, work as a specialist).

**Table 2:** The comparison of historical data and the simulated number of physicians

Starting year	**1984**	**1993**
**Target year**	1991	2011
**Historical data**	9303	12,532
**Simulated data**	9480	12,465
**Relative error**	0.019	0.005

**Scenario 1:** The existing enrollment of 590 students per year and an average of 450 new ST residents starting ST per year (historical average from 2006 to 2016).

**Figure 3:**
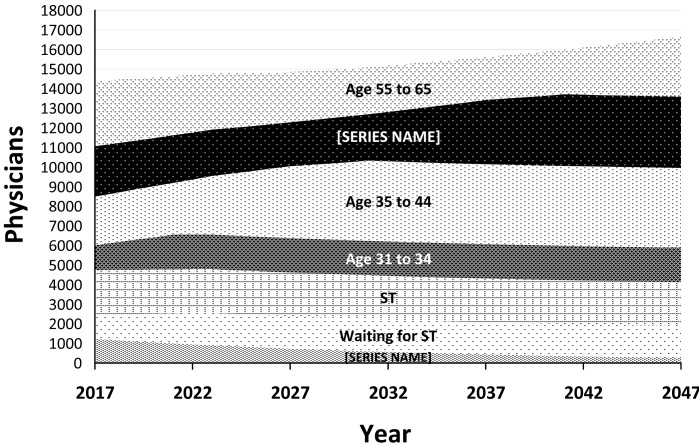
Age distribution of medical specialists in Croatia according to Scenario 1.

In 2017 the Croatian public health workforce comprised 14 394 physicians (346 per 100 000 inhabitants). Our simulation projected that by 2021 this figure would reach 14 637 (353 per 100 000 inhabitants), meaning that the number of physicians will certainly reach the current EU average of 360 per 100 000 inhabitants and will continue to increase. Furthermore, there will be 15 098 physicians (381 per 100 000 inhabitants) by 2031 and 15 998 (430 per 100 000 inhabitants) by 2041. This scenario showed a drop out of 3737 physicians over 30 years or on average 125 yearly, mostly due to emigration, career-ending disability, and mortality. The model predicted improvements in the age distribution of specialists, with an increasing number of younger specialists and a decreasing number of older ones (Figure 3).

**Scenario 2:** The existing enrollment of 590 medical students per year, with an average of 550 new ST residents starting ST per year (the Ministry of Health of the Republic of Croatia strived for this in 2017 and 2018).

**Figure 4:**
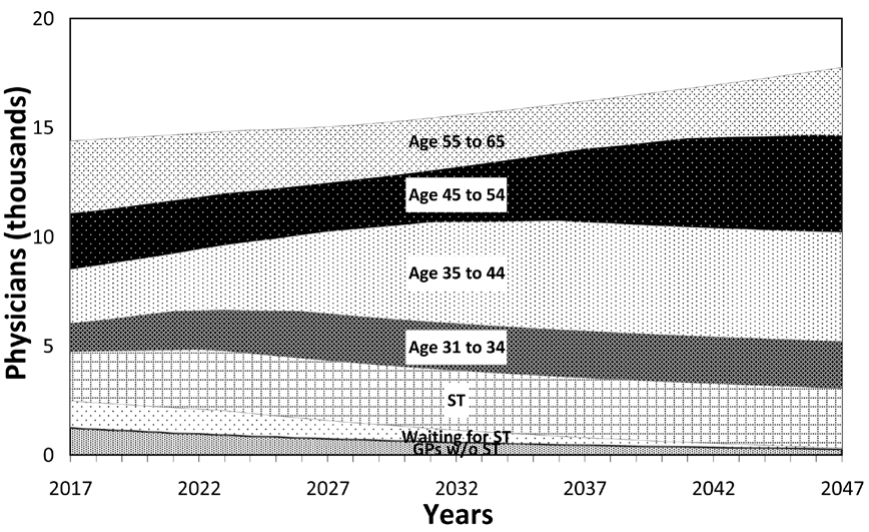
Age distribution of medical specialists in Croatia according to Scenario 2.

**Figure 5:**
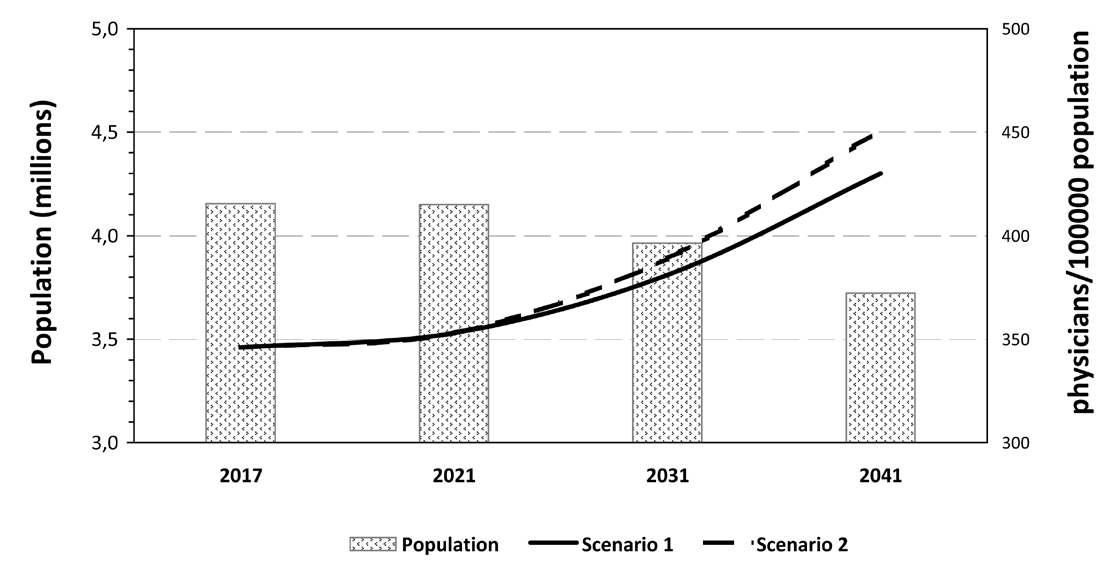
Comparison of population projections and physician trends according to scenarios.

By 2021, the number of physicians would reach 14 665 (353 per 100 000 inhabitants). Similarly to Scenario 1, this also represents an increase in the number of physicians from 2017 to 2021, with Croatia certainly reaching the current EU average. By 2031, the number of physicians will be 15 428 (389 per 100 000 inhabitants); by 2041, it will be 16 792 (451 per 100 000 inhabitants). This scenario included a drop out of 4061 physicians over 30 years or on average 135 yearly. The age distribution of physicians is shown in Figure 4. A comparison of the two simulated scenarios is shown in Figure 5.

The two presented scenarios showed that Croatia would not face a physician shortage in the future. Both scenarios showed that the Croatian physicians’ age distribution would recover over time in favor of younger age groups of specialists.

## Discussion

The constructed model replicates the flow of physicians’ population from medical school enrollment till retirement. It forecasts the supply and age distribution of physicians in Croatia. The model shows that the number of practicing physicians in Croatia will continue to increase until 2041. Different scenarios simulate possible future situations under changing assumptions and can be used to help stakeholders and policymakers in decision-making (19).

The average number of physicians per capita in the most developed EU countries was chosen as a target and Croatia aspires to reach this number. The total number of physicians shows an increasing trend, but technological development in the field of medicine and skill mix and task shift to other health professionals must be taken into account. In many countries, physicians no longer have monopoly over certain medical treatments, and the functions of other qualified health care professionals have been expanded (20).

The two presented scenarios indicate that Croatia currently has a sufficient number of physicians. There is no need to adjust medical school enrollment, which was last increased in 2009. With the current enrollment policy, in the coming years Croatia could face a surplus of physicians already seen in the past (14,21).

Since Croatia accessed the EU in 2013, around 800 physicians have left the country (approximately 130 per year). However, judging from the experience of other Member States that joined the EU in 2004 or later, this might have been the peak of the emigration wave due to EU accession, and emigration is expected to decrease and stabilize in the following years (22).

Croatia has 29.4% physicians aged 55 years and over. This is an acceptable rate, especially if we compare it with the rates in other countries: Italy (55%), Bulgaria (50.8%), Estonia (45.9%), France (45.1%), Germany (44.6%), Hungary (43.4%), Spain (34.1%), Sweden (33.7%), Denmark (32.5%), Austria (29.7%), and Slovenia (29.5%) (4).

Both scenarios simulated in this study indicate that the Croatian physicians’ age distribution will recover over time in favor of younger age groups of specialists. A prerequisite is that the Croatian Ministry of Health continues to issue licenses for ST to produce specialists on time. These results should be considered in the context of the current pessimistic demographic projections for the Croatian population. However, if this trend decreases or is reversed because of increased fertility, immigration, or other reasons, the average number of physicians in the future might be closer to the numbers observed in the EU.

There is a large urban-rural disparity and regional differences in the density of physicians, especially specialists. The highest density of physicians is in regional centers (Zagreb, Split, Rijeka, Osijek), which are also the seats of medical schools. Other research groups also used SD modeling to forecast the demand and supply of medical workforce at the regional or national level (8-10,19). Proactive planning and management of the health care workforce, including continued monitoring, might facilitate appropriate and necessary adjustments to the supply side. SD simulation modeling may be used to present and analyze many public health issues and challenges (23), especially since it has an important advantage of parameter modification. The model allows the researchers to change the elements in a fairly simple way and assess how the changes affect the final results.

The study has several limitations. We did not consider the number of medical students in foreign study programs run by Croatian medical schools. We also did not take into account geographical/regional distribution of physicians and distribution according to the health care levels, as well as technological advances that may affect the demand for physicians. A significant limitation is the lack of normative requirements for the demand of specialists. Future research and models need to be more specific and include individual medical specialties with full-time equivalent. Contrary to the results of this research, our previous study predicted a serious future shortage of physicians (18). At that time, the Croatia Bureau of Statistics data from 2011 were used as input data for the Croatian population projections. Since these projections were shown to be inaccurate by the yearly reports of population migration, in the present study we used the corrected population forecast recently adjusted by leading demographic experts (17).

In conclusion, Croatia has enough physicians per capita, and there is no need to either increase the medical school enrollment or establish new medical schools (24). It is necessary to keep the recently reached level of 550 licenses for ST per year because the current development of medicine requires mainly specialists. By allowing physicians to begin ST at the right time, we prevent and reduce the physicians’ emigration. System dynamics methods proved to be suitable for modeling physicians’ dynamics, including their study, ST training, migration, and aging and to forecast their number and age structure under different scenarios. A developed model is made available online and can be freely used or adapted to other health care systems and circumstances.
